# Curative-intent lung resection after recovery from fulminant immune checkpoint inhibitor-associated myocarditis during neoadjuvant therapy: a case report

**DOI:** 10.1186/s44215-026-00278-x

**Published:** 2026-10-09

**Authors:** Yui Kaburaki, Hiroyuki Ito, Tetsuya Isaka, Takashi Tsunematsu, Kengo Tsukahara, Azusa Hayakawa, Ikki Takada, Ryotaro Matsuyama, Chiaki Kanno, Takuya Nagashima

**Affiliations:** 1 Department of Thoracic Surgery, Kanagawa Cancer Center, 2-3-2 Nakao, Asahi-Ku, Yokohama, Kanagawa 241-8515 Japan; 2 Department of Cardiology, Kanagawa Cancer Center, 2-3-2 Nakao, Asahi-Ku, Yokohama, Kanagawa 241-8515 Japan; 3 Division of Cardiology, Fujisawa City Hospital, 2-6-1 Fujisawa, Fujisawa, Kanagawa 251-8550 Japan; 4 Department of Cardiology, Yokohama Brain and Spine Center, 1-2-1 Takigashira, Isogo-Ku, Yokohama, Kanagawa 235-0012 Japan

**Keywords:** Immune checkpoint inhibitor, Myocarditis, Non-small-cell lung cancer, Sleeve resection, Pulmonary artery reconstruction, Neoadjuvant chemoimmunotherapy

## Abstract

**Background:**

Immune checkpoint inhibitor (ICI)-associated myocarditis is a rare but potentially fatal immune-related adverse event (irAE) that may interrupt planned curative treatment for resectable non-small-cell lung cancer. However, the suitability of definitive surgery after recovery from fulminant myocarditis remains unclear.

**Case presentation:**

A 53-year-old man with left upper lobe adenocarcinoma and ipsilateral hilar nodal disease was administered four cycles of neoadjuvant cisplatin, pemetrexed, and pembrolizumab, resulting in a partial response. Eighty-six days after the initiation of neoadjuvant chemoimmunotherapy, he developed grade 4 fulminant ICI-associated myocarditis with ventricular fibrillation and cardiac arrest. Endomyocardial biopsy revealed active lymphocytic myocarditis. The patient required intubation, percutaneous cardiopulmonary support, intra-aortic balloon pumping, continuous hemodiafiltration, high-dose corticosteroids, and prolonged rehabilitation. After his cardiac function recovered, tumor regrowth without distant metastasis was identified. Multidisciplinary reassessment supported surgery because the left ventricular ejection fraction had recovered to 71%, no recurrent malignant arrhythmia had been documented, cardiac biomarkers had improved, prednisolone had been tapered to 10 mg/day, and the disease remained technically resectable. Thoracoscopic-assisted thoracotomy with left upper lobe sleeve resection, pulmonary artery reconstruction, and hilar and mediastinal lymph node dissection was performed 144 days after myocarditis onset, corresponding to 230 days after the initiation of neoadjuvant chemoimmunotherapy. R0 resection was achieved. Surveillance computed tomography showed no evidence of recurrence through 3.5 years after surgery, and the patient was alive at the last follow-up 6.3 years after surgery.

**Conclusions:**

Fulminant ICI-associated myocarditis does not necessarily preclude subsequent curative-intent lung resection. In highly selected patients, surgery may remain feasible after objective cardiac recovery, multidisciplinary reassessment, and shared decision-making with the patient. This case suggests that the acute management of life-threatening cardiovascular irAEs should be undertaken at centers with continuous on-site cardiology support, multidisciplinary care, and access to emergency mechanical circulatory support. Additionally, planning subsequent curative-intent surgery requires multidisciplinary collaboration, ready access to cardiology expertise, and an appropriate pathway for rapid escalation of cardiovascular care.

## Background

Perioperative pembrolizumab plus platinum-based chemotherapy improves outcomes in resectable non-small-cell lung cancer (NSCLC) [1]. However, severe immune-related adverse events (irAEs) can interrupt planned curative treatment. Immune checkpoint inhibitor (ICI)-associated myocarditis is uncommon but potentially fatal and requires prompt recognition, high-dose corticosteroid therapy, intensive monitoring, and escalation of treatment when indicated [2–5].

Evidence regarding definitive surgery after fulminant ICI-associated myocarditis remains limited. Published reports on ICI-associated myocarditis have mainly focused on diagnosis, acute management, and prognosis, rather than subsequent local curative treatment [6–9]. Here, we describe the practical reassessment process that enabled curative-intent lung resection after recovery from life-threatening myocarditis complicated by ventricular fibrillation and cardiac arrest in a patient with left upper lobe adenocarcinoma with ipsilateral hilar nodal disease.

## Case presentation

**Fig. 1:**
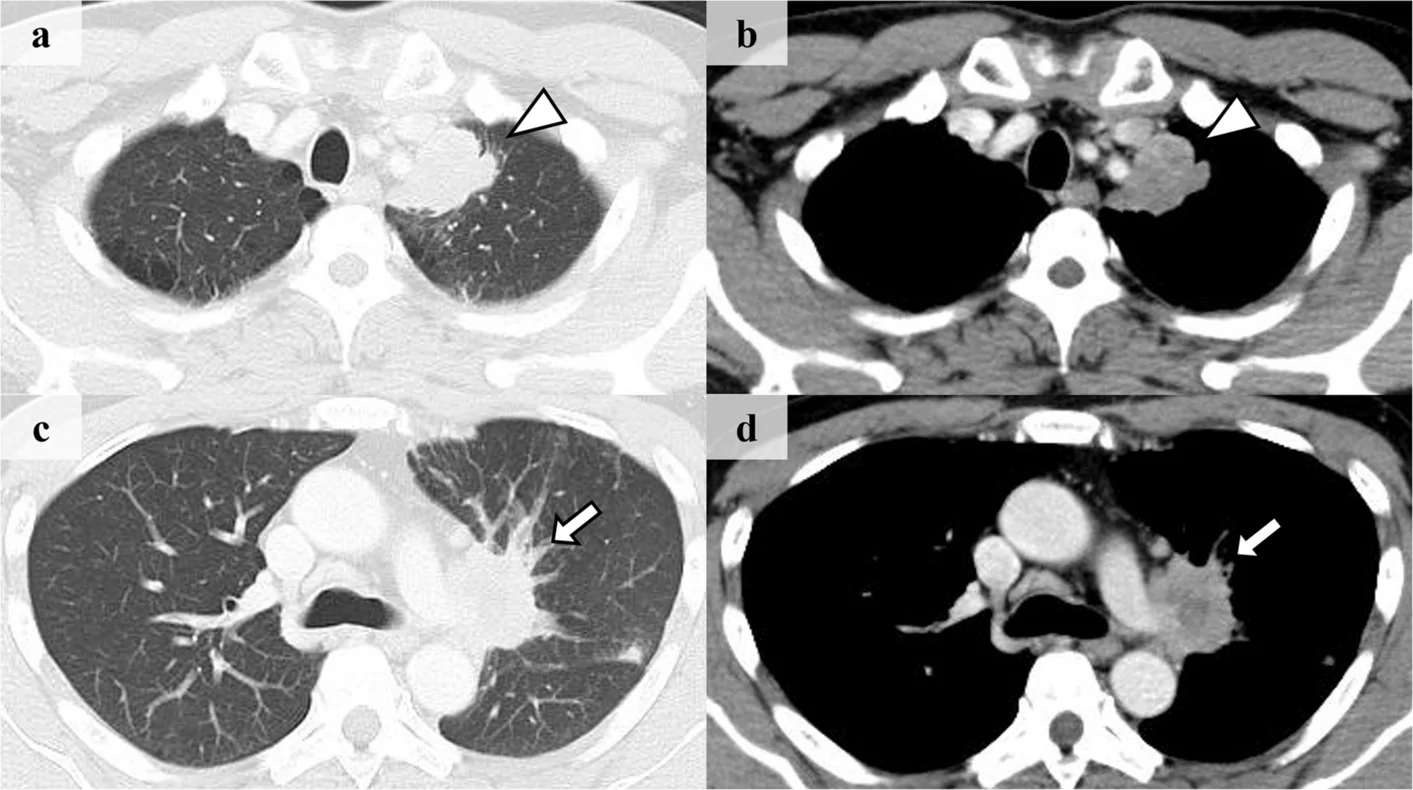
Baseline CT images (before neoadjuvant chemoimmunotherapy). Axial CT images show the left apical primary tumor (arrowheads; longest diameter, 38 mm) and left hilar lymph node enlargement (arrows; short-axis diameter, 38 mm). **a** Lung window, primary tumor; **b** mediastinal window, primary tumor; **c** lung window, hilar lymph node; **d** mediastinal window, hilar lymph node

**Fig. 2:**
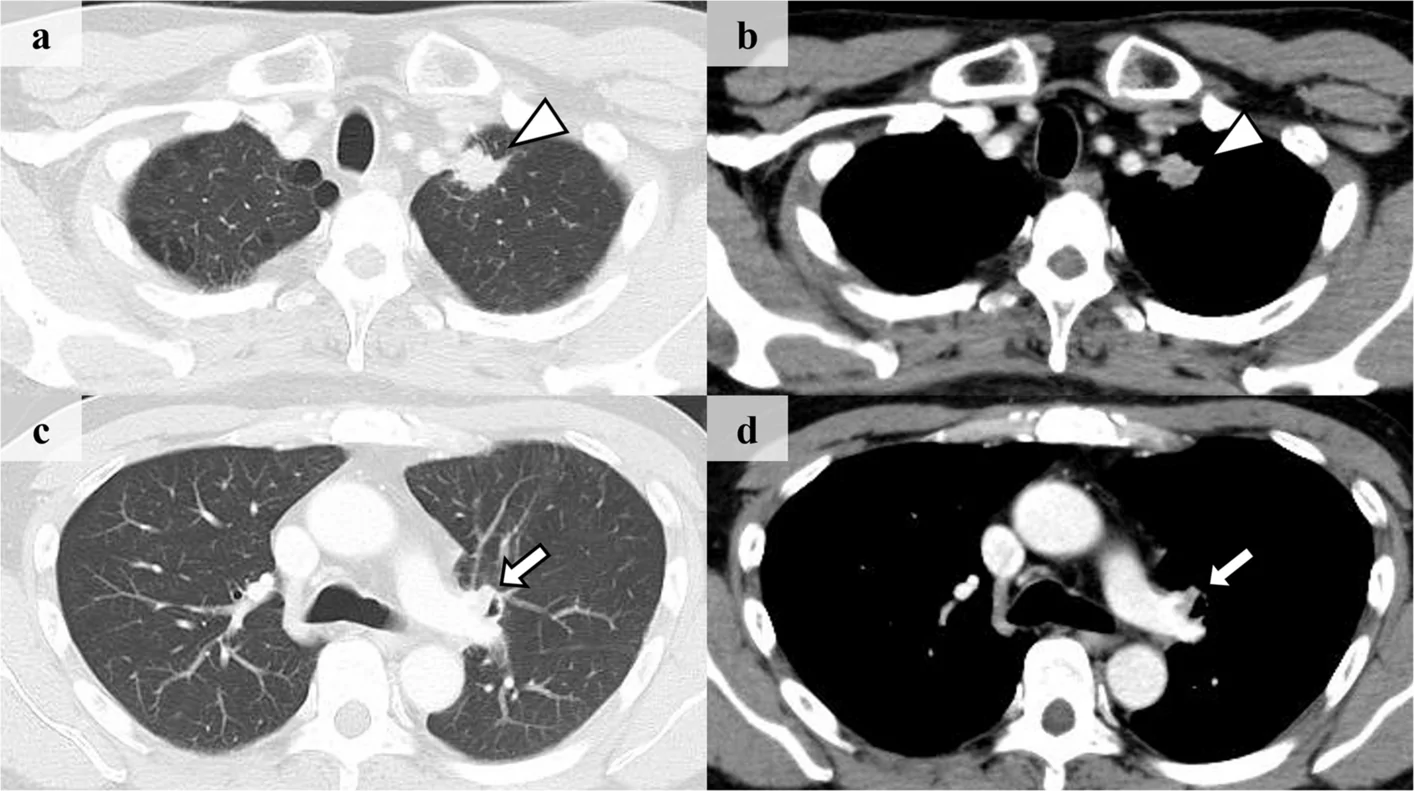
Post-neoadjuvant chemoimmunotherapy CT images showing partial response. Axial CT images obtained after neoadjuvant chemoimmunotherapy show reduction of the left apical primary tumor (arrowheads; longest diameter, 14 mm) and left hilar lymph node (arrows; short-axis diameter, 5 mm), corresponding to a partial response based on Response Evaluation Criteria in Solid Tumors. **a** Lung window, primary tumor; **b** mediastinal window, primary tumor; **c** lung window, hilar lymph node; **d** mediastinal window, hilar lymph node

A 53-year-old man was diagnosed with left upper lobe adenocarcinoma with ipsilateral hilar nodal disease (cT2aN1M0, stage IIB), and further examination showed a programmed death-ligand 1 (PD-L1) tumor proportion score < 1%. Oncomine testing showed no actionable alterations in *EGFR*, *ALK*, *ROS1*, or other tested genes. The patient was administered four cycles of neoadjuvant cisplatin, pemetrexed, and pembrolizumab according to the KEYNOTE-671 protocol. Subsequently, the primary tumor diameter decreased from 38 to 14 mm, while the short-axis diameter of the hilar node decreased from 38 to 5 mm (Figs. 1 and 2). These changes constituted a partial response according to the Response Evaluation Criteria in Solid Tumors.

**Fig. 3:**
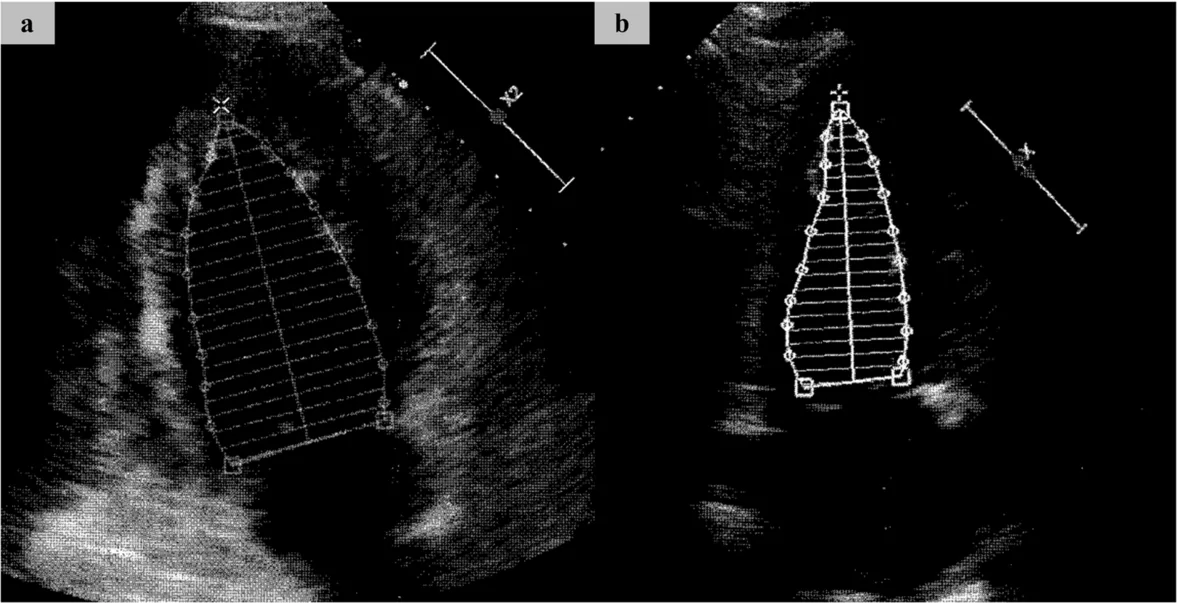
Echocardiographic recovery after ICI-associated myocarditis. Representative end-systolic apical two-chamber images at (**a**) myocarditis onset and (**b**) before surgery show recovery of left ventricular systolic function. LVEF increased from 51% at onset to 71% before surgery. At onset, diffuse left ventricular hypokinesis was most pronounced in the anterior wall. A trace pericardial effusion was present

Eighty-six days after neoadjuvant chemoimmunotherapy initiation (approximately 3 weeks after the last cycle), the patient developed dyspnea and cough and was admitted for preoperative evaluation. Initial quantitative echocardiography showed a left ventricular ejection fraction (LVEF) of 51% (Fig. 3a). Subsequently, his condition deteriorated, with 87/60 mmHg blood pressure, 120 beats/min heart rate, and 89% oxygen saturation on room air. Repeat echocardiography showed diffuse severe left ventricular hypokinesis with a visually estimated LVEF of approximately 20%, which subsequently decreased to approximately 10% during the early critical phase. Electrocardiography showed low voltage in the limb leads, Q waves in V1–V4, and ST-segment elevation in leads I, aVL, and V1–V4. Chest radiography showed bilateral pulmonary congestion. His initial creatine kinase, creatine kinase-MB, N-terminal pro-B-type natriuretic peptide, and troponin T levels were 966 U/L, 90 U/L, 9,388 pg/mL, and 5.84 ng/mL, respectively. Coronary angiography showed no significant coronary stenosis. Further, endomyocardial biopsy showed active lymphocytic myocarditis without significant fibrosis, with focal infiltrates predominantly composed of CD3-positive T lymphocytes (CD8 > CD4) and macrophages.

**Fig. 4:**
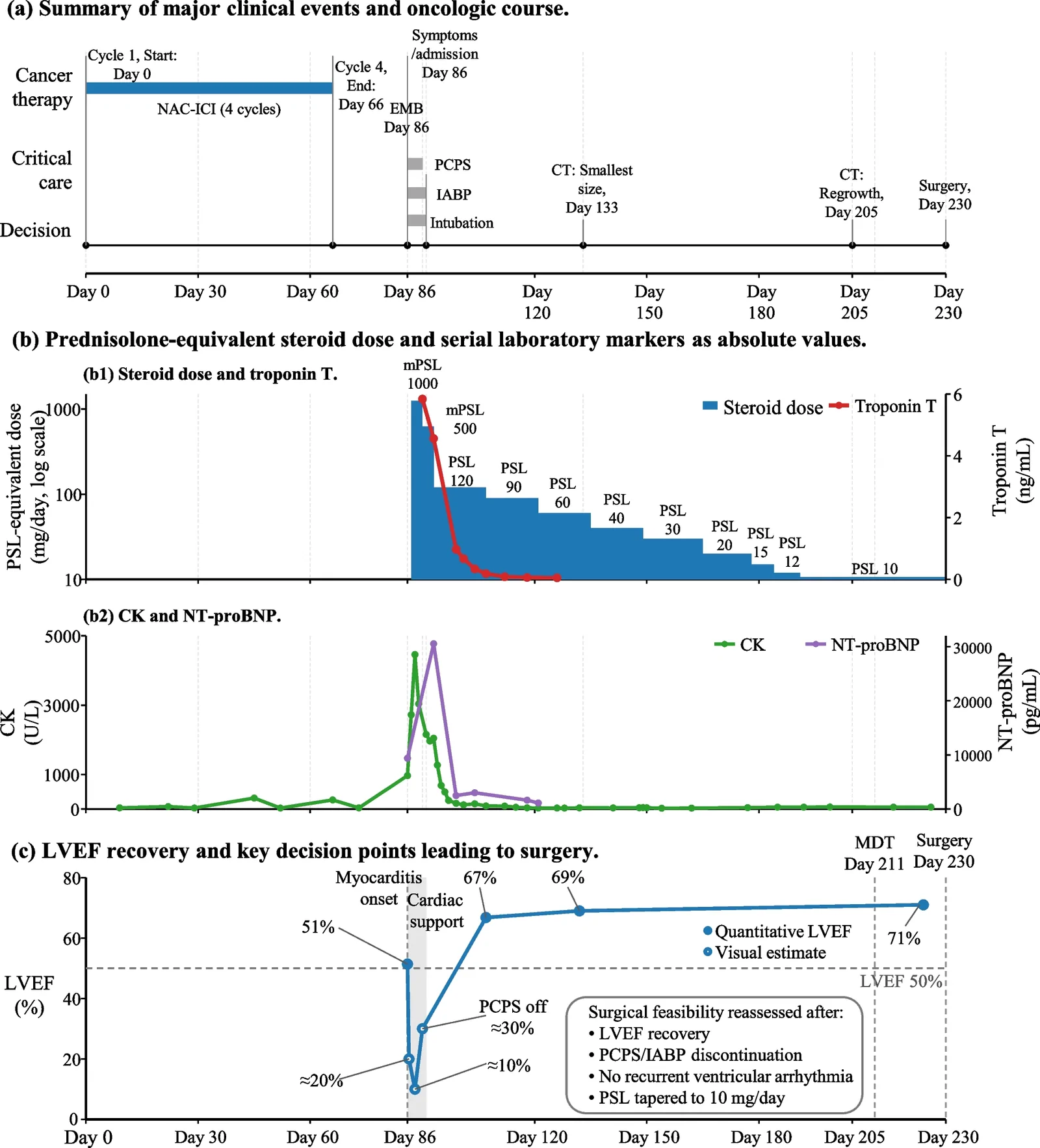
Clinical timeline from neoadjuvant chemoimmunotherapy to surgery. Day 0 was defined as the first day of neoadjuvant chemoimmunotherapy. **a** Summary of major oncologic and cardiovascular events, **b** Prednisolone-equivalent steroid dose and serial laboratory markers as absolute values: steroid dose and troponin T (b1), and CK and NT-proBNP (b2). **c** LVEF recovery and key decision points leading to surgery. Methylprednisolone doses were converted to prednisolone-equivalent doses using methylprednisolone 4 mg = prednisolone 5 mg. CT on day 205 showed tumor regrowth without distant metastasis. The filled and open circles indicate quantitatively measured and visually estimated LVEF values, respectively. Abbreviations: CK, creatine kinase; CT, computed tomography; EMB, endomyocardial biopsy; IABP, intra-aortic balloon pumping; ICI, immune checkpoint inhibitor; LVEF, left ventricular ejection fraction; MDT, multidisciplinary team; mPSL, methylprednisolone; NAC-ICI, neoadjuvant chemoimmunotherapy; NT-proBNP, N-terminal pro-B-type natriuretic peptide; PCPS, percutaneous cardiopulmonary support; PSL, prednisolone; filled circles, quantitative LVEF; open circles, visual estimate

During evaluation, ventricular fibrillation led to cardiac arrest requiring intubation, percutaneous cardiopulmonary support, intra-aortic balloon pumping (IABP), continuous hemodiafiltration, and intensive care. Because the event was life-threatening and required urgent mechanical circulatory and respiratory support, it was classified as grade 4 myocarditis according to the Common Terminology Criteria for Adverse Events. Methylprednisolone was administered at 1,000 mg/day for 3 days and at 500 mg/day for the next 3 days, followed by oral prednisolone initiated at 120 mg/day and gradually tapered based on his clinical course and cardiac function recovery. By the time of surgery, prednisolone was reduced to 10 mg/day. Percutaneous cardiopulmonary support was discontinued 4 days after myocarditis onset, and IABP and mechanical ventilation were discontinued the following day. The course was complicated by transient renal failure, deep venous thrombosis, and lower-extremity neurologic deficits requiring rehabilitation. Spinal cord infarction was suspected, but the final cause of the neurologic deficits remained undetermined. The clinical course is summarized in Fig. 4.

Cardiac function gradually recovered, with LVEF improving to 67%, 69%, and 71% on days 107, 132, and 224, respectively, after neoadjuvant chemoimmunotherapy initiation (Fig. 3b). No recurrent malignant arrhythmia was documented after the withdrawal of circulatory support. Follow-up computed tomography (CT) obtained 205 days after neoadjuvant chemoimmunotherapy initiation showed tumor regrowth without distant metastasis. At preoperative reassessment, the maximum diameter of the primary lesion was 2.1 cm, including a 1.8-cm solid component. Hilar nodal disease persisted at station 12u. Despite impaired activities of daily living, preoperative electrocardiography and echocardiography were unremarkable, and lower-extremity venous ultrasonography showed no residual thrombosis. After multidisciplinary review of oncologic need, resectability, alternatives, and perioperative risk, the cardiology team considered surgery feasible but recommended close monitoring for recurrent ventricular arrhythmia. After the risks were explained, the patient elected surgery.

Thoracoscopic-assisted thoracotomy was performed 144 days after myocarditis onset (230 days after neoadjuvant chemoimmunotherapy initiation) and included left upper lobe sleeve resection, pulmonary artery reconstruction, and hilar and mediastinal lymph node dissection. The operative time was 6 h 3 min, blood loss was 590 mL, and a minor air leak resolved with conservative management. No perioperative cardiovascular events occurred. Pathology showed ypT3N1M0 adenocarcinoma with negative margins (R0) and minor treatment effect (Ef 1a). T3 was assigned because multiple separate tumor nodules, measuring up to 8 mm, were present in the same lobe. Pembrolizumab was permanently discontinued after grade 4 myocarditis, and no adjuvant systemic therapy was administered. Surveillance CT showed no evidence of recurrence through 3.5 years after surgery. The patient was alive at the last follow-up, 6.3 years after surgery.

## Discussion and conclusions

Perioperative pembrolizumab combined with platinum-based chemotherapy improves outcomes in resectable NSCLC [1]. However, severe irAEs can interrupt the intended perioperative pathway. ICI-associated myocarditis is uncommon, but its reported incidence varies according to treatment regimen, surveillance intensity, and case definition. In a systematic review and meta-analysis of 589 clinical trials including 83,315 patients, the reported all-grade rates of myocarditis with PD-1 monotherapy, PD-L1 monotherapy, ipilimumab, and dual ICI therapy were 0.24%, 0.33%, 0.15%, and 0.72%, respectively [6]. In contrast, the surveillance of a real-world cohort using serial biomarkers revealed ICI-associated myocarditis in 27 of 2606 patients (1.0%) [7].

Despite its low incidence, clinically recognized ICI-associated myocarditis can be severe or fatal. In a multicenter registry, major adverse cardiac events occurred in 46% of patients with ICI-associated myocarditis [2]. A recent systematic review also reported myocarditis-associated mortality in 37.7% of published cases [6].

Evidence regarding late recurrence of ICI-associated myocarditis after clinical recovery without ICI rechallenge remains limited. Current irAE guidance recommends close clinical evaluation during corticosteroid tapering because of the risk of irAE rebound or recurrence [3]. A recent report of rechallenge after severe ICI-associated myocarditis showed myocarditis recurrence in 37.5% of patients, although this estimate was based on a small sample size (*n*= 16) [9]. However, these data were obtained in ICI rechallenge settings and therefore differ from the present case, in which ICI therapy was not resumed.

This case illustrates that surgery may remain an option in highly selected patients when cardiac recovery, oncologic urgency, technical resectability, and patient preference are carefully aligned. Current cardio-oncology and irAE guidelines recommend prompt recognition, high-dose corticosteroids, intensive monitoring, and treatment escalation for suspected ICI-associated myocarditis, but do not define validated criteria for resuming elective major thoracic surgery after fulminant myocarditis [3, 4]. In this case, operative candidacy was reassessed based on LVEF recovery to 71%, withdrawal from mechanical circulatory support, absence of recurrent ventricular arrhythmia, improved cardiac biomarkers, prednisolone tapering to 10 mg/day, absence of distant metastasis, and preserved technical resectability.

The decision to proceed with surgery after fulminant myocarditis remains controversial. Although definitive radiotherapy or chemoradiotherapy was also considered, surgery was selected because the disease remained localized and technically resectable after objective cardiac function recovery. The residual risk of recurrent ventricular arrhythmia and the need for close perioperative cardiac monitoring were discussed with the patient.

Life-threatening ICI-associated myocarditis should be managed at a center with continuous on-site cardiology, multidisciplinary support, and access to emergency mechanical circulatory assistance. Once cardiac function had normalized and ventricular arrhythmias were absent, the cardiology team considered surgery feasible within an individualized perioperative plan. Nevertheless, ventricular arrhythmias may still occur after apparent recovery; therefore, perioperative planning should include continuous electrocardiographic monitoring, immediate defibrillation capability, and prompt cardiology support.

This clinical course should not be regarded as a standard implementation of the KEYNOTE-671 perioperative strategy. Fulminant myocarditis caused prolonged interruption of the intended perioperative schedule, and surgery was performed 144 days after myocarditis onset. Therefore, the present case should be preferably interpreted as an individualized, nonstandard pathway to curative-intent surgery after severe immune-related toxicity.

Evidence regarding surgery after preoperative irAEs remains limited. A multicenter retrospective analysis of resectable NSCLC treated with neoadjuvant nivolumab plus chemotherapy reported that preoperative irAEs occurred in approximately 20% of patients and were associated with treatment discontinuation and surgery cancellation, although myocarditis-specific outcomes were not separately defined [10]. A perioperative case report described fatal, unrecognized ICI-associated myocarditis in a patient with metastatic NSCLC presenting for mediport placement, underscoring the perioperative risk of occult myocarditis [8]. Unlike these reports, myocarditis in the present case was diagnosed, treated, and objectively reassessed before curative-intent lung resection.

This case does not establish general criteria for operative timing. In highly selected patients, curative-intent surgery may remain an option when cardiac recovery, oncologic need, technical resectability, and patient preference align. Acute management of life-threatening cardiovascular irAEs should be undertaken at centers with continuous on-site cardiology support, multidisciplinary care, and access to emergency mechanical circulatory support. Planning subsequent curative-intent surgery requires multidisciplinary collaboration, ready access to cardiology expertise, and an appropriate pathway for rapid escalation of cardiovascular care.

## Data Availability

All data generated or analyzed during this study are included in this published article. Additional de-identified details are not publicly available due to patient privacy considerations.

## Abbreviations

CT: Computed tomography
IABP: Intra-aortic balloon pumping
ICI: Immune checkpoint inhibitor
irAE: Immune-related adverse event
LVEF: Left ventricular ejection fraction
NSCLC: Non-small-cell lung cancer
PD-L1: Programmed death-ligand 1
